# Cardiac Function and Outcome in Patients with Cardio-Embolic Stroke

**DOI:** 10.1371/journal.pone.0095277

**Published:** 2014-04-23

**Authors:** Jung-Ick Byun, Keun-Hwa Jung, Young-Dae Kim, Jeong-Min Kim, Jae-Kyu Roh

**Affiliations:** 1 Department of Neurology, Clinical Research Institute, Seoul National University Hospital, Seoul, South Korea; Program in Neuroscience, Seoul National University College of Medicine, Seoul, South Korea; 2 Department of Neurology, Yonsei University College of Medicine, Seoul, Korea; 3 Department of Neurology, ChungAng university hospital, Seoul, Korea; 4 Department of Neurology, Armed Forces Capital Hospital, Seongnam, Gyeunggido, South Korea; S.G.Battista Hospital, Italy

## Abstract

**Background:**

The relationship between whole spectrum of Ejection fraction (EF) and cardioembolic stroke (CES) outcome has not been fully described yet. Notably, it remains unclear whether borderline EF (41∼49%) is related with poor outcome after CES. We sought to evaluate whether lower ejection fraction and borderline EF could predict the outcome in patients with CES.

**Method and Results:**

We evaluated the relationship between EF and functional outcome in 437 consecutive patients with CES. EF was introduced as continuous and categorical (EF≤40%, EF 41∼49%, EF≥50%) variable. Patients with CES and the subgroup with AF were evaluated separately. Poor short-term outcome (modified Rankin Score≥3at discharge or death within 90 days after stroke onset) and long-term mortality were evaluated. A total of 165 patients (37.8%) had poor short-term outcomes. EF tends to be lower in patients with poor short-term outcome (56.8±11.0 vs. 54.8±12.0, p-value 0.086). Overall cumulative death was136 (31.1%) in all CES patients and 106 (31.7%) in the AF subgroup. In a multivariable model adjusted for possible covariates, the hazard ratio for mortality significantly decreased by 3% for every 1% increase in ejection fraction in CES patients and 2% for every 1% increase in the AF subgroup. Reduced EF (EF≤40%) showed higher mortality (HR 2.61), and those with borderline EF (41∼49%) had a tendency of higher mortality (HR 1.65, p-value 0.067)compared with those with normal EF.

**Conclusion:**

We found a strong association between lower EF and CES outcome. Echocardiographic evaluation helps to better determine the prognosis in CES patients, even in subgroup of patients with AF.

## Introduction

Heart disease is a major risk factor for stroke following age and hypertension [1]. Ejection fraction (EF), the proportion of left ventricular volume emptied during ventricular systole, is a reliable measure of left ventricular (LV) systolic function [2]. Reduced EF provides objective documentation of cardiac dysfunction [3].Previous studies identified low EF as a predictor of mortality in stroke patients [4]–[6]. However, there are limitations in the previous studies. First, EF was arbitrarily dichotomized and evaluated as a categorical variable. In consequence, the relationship between full spectrum of EF and stroke outcome was not properly evaluated, especially those with borderline EF. Second, previous studies evaluated stroke patients regardless of its subtype. Cardioembolic stroke (CES) is known to cause more severe stroke [7], [8] and higher mortality [9], [10] than other stroke subtypes. Moreover, atrial fibrillation (AF), the most common source of CES [11], is known to be independently associated with higher case fatality in CES. Decreased EF may have more impact on CES outcome, especially on those with AF. This study attempted to address this shortcoming by exploring the influence of EF as continuous and categorical variable in patients with CES. We sought to evaluate whether lower ejection fraction and borderline EF could predict outcome in patient with CES and in subgroup with AF.

## Methods

### Ethics statement

The guidelines and procedures of this registry were approved by the Institutional Review Board of the Seoul National University Hospital. Written informed consent to participate was obtained from all patients or their next of kin if the inclusion criteria were met.

### Study population

We performed a retrospective cohort study based of consecutive patients with CES. Among acute stroke patients consecutively registered in the Seoul National University Hospital Stroke Registry (SNUHSR) between January 2002 and December 2010, those with cardioembolism subtype were included in this study. Patients with transient ischaemic symptom or those without documented EF measurement were excluded. The SNUHSR is a prospective hospital based registry for patients with acute ischemic stroke or transient ischemic attack admitted within 7 days after symptom onset. The registry dataset included information of demographics, stroke subtypes, vascular risk factors, investigations performed in the hospital and neurological outcomes at discharge. Clinical, radiological and laboratory evaluations allowed us to disclose the stroke etiology and the diagnosis of CES was made according to the Trial of ORG10172 in Acute Stroke Treatment (TOAST) classification [12].

### Vascular risk factors and cardiac evaluation

We collected demographics, vascular risk factors and in- hospital evaluation and management for each patient. Vascular risk factors included history of previous stroke, hypertension, diabetes mellitus, hyperlipidemia and smoking. Hypertension was defined as a systolic blood pressure >140 mm Hg, a diastolic blood pressure >90 mm Hg and/or current use of anti-hypertensive agents. Diabetes mellitus was diagnosed by the relevant clinical or drug history or the biochemical evidence of at least two measurements of fasting blood glucose readings >7 mmol/L. Dyslipidemia was defined as current use of lipid-lowering agents or at least two elevated serum lipid measurements (total cholesterol >6.2 mmol/L or low-density lipoprotein-cholesterol >4.1 mmol/L). Smoking was defined as tobacco use within 1 month of admission [13]. Hemorrhagic transformation was documented when initial or follow-up brain imaging revealed hemorrhage on the site of ischemic stroke. The severity of the stroke was evaluated by a trained neurologist using National Institute of Health Stroke Scale (NIHSS) and disability was accessed using modified Rankin score (mRS) at admission and at discharge. NIHSS is a 11-item neurologic examination stroke scale for use in acute stroke therapy trials [14].The modified Rankin scale is a commonly used scale measuring disability or dependence in activities of daily life in stroke patients [15].

Standard cardiac evaluation included 12-lead electrocardiography (ECG)and transthoracic echocardiography. Holter monitoring or transesophageal echocardiography was performed when standard cardiac evaluation did not reveal any cardioembolic source in high clinical suspicion of CES. Diagnosis of AF was made when there was a documented history of paroxysmal AF or ECG data showing AF during hospitalization. Both chronic and paroxysmal AF was included. Echocardiogram was performed with the patient resting supine in the left lateral position during hospital admission and interpreted by a trained cardiologist. EF was calculated by biplane modified Simpson's method [16].

### Outcome parameters

Poor short-term functional outcome and long-term mortality were evaluated. Poor short-term outcome was characterized by mRS≥3at discharge or death within 90 days after stroke onset. Mortality data were obtained from the Korean National Vital Statistics system with currency of December 2010. In Korea, all deaths must be reported to the National Vital Statistical Office by law and the data are known to be reliable [17].

### Statistical analysis

Statistical analysis was performed to evaluate the relationship between EF and outcome parameters. Patients with CES and the subgroup with AF were evaluated separately. All data in this study are expressed as mean ± standard deviations (SD) or numbers (percentages). Continuous variables were tested for normal distribution with the Kolmogorov-Smirnov test. Mann-Whitney U test was used to compare EF according to short-term functional outcome. EF according to mRS and 90 days mortality was separately analyzed using Kruskal-Wallis one-way analysis of variance and Mann-Whitney U test respectively. Predictors for outcome were determined by multivariate logistic regression analysis. All variables except for age, EF and NIHSS were dichotomized. EF was first introduced as continuous variable. And we divided EF into three groups (≤40%, 41∼49%, >50%) and introduced it as a categorical variableto further evaluate the relationship between EF and CES outcome. Age was introduced as continuous variable and NIHSS was categorized into three groups following the usual criteria [18]: NIHSS score <7, NIHSS 7–14 and NIHSS score >14. Comparisons between groups were analyzed as follows: continuous variables by Student's t-test (2-sided) or Mann-Whitney U test and categorical variables by Pearson's chi square test. Variables with p-value <0.1 in univariate analysis were entered into the multivariate model.

To identify relationship between EF and long-term mortality, we first used multivariate Cox regression model introducing EF as a continuous variable. The model adjusted possible predictors of mortality and morbidity including age, sex, previous history of stroke, hypertension, diabetes, dyslipidemia, smoking, NIHSS at admission, intra-venous or intra-arterial thrombolysis, hemorrhagic transformation and warfarin at discharge. To further evaluate the relationship between EF and long-term mortality, we divided the range of EF into 3 groups as previously described: normal (>50%), borderline (41–49) and reduced (≤40%) [3]. Kaplan-Meier analysis was used to estimate survival condition and the log-rank test was used to compare rate estimates according to EF. The same Cox regression model was applied to evaluate relationship between long-term mortality and the three EF groups. The variables were cross tabulated to discard multicollinearity. Probability values of <0.05 were considered statistically significant. SPSS software (SPSS 12.0, SPSS Inc., Chicago, IL, USA) was used for the analysis.

## Results

### Patient characteristics and cardiac sources of embolism

**Figure 1:**
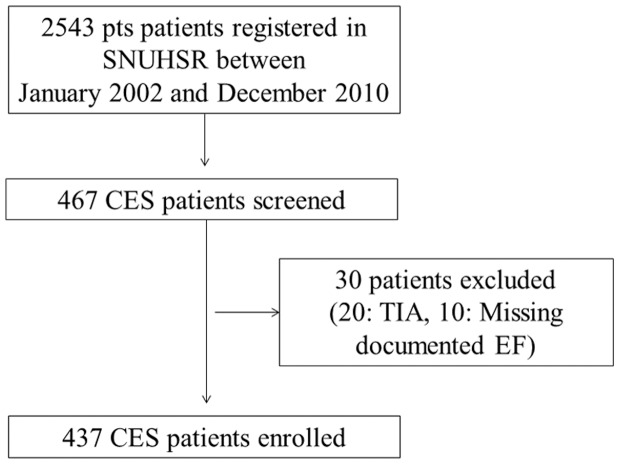
Flow chart of patient enrollment. Abbreviations: SNUHSR: Seoul National University Hospital Stroke Registry, CES: Cardio-embolic stroke, TIA: Transient ischemic attack, EF: Ejection Fraction.

**Figure 2:**
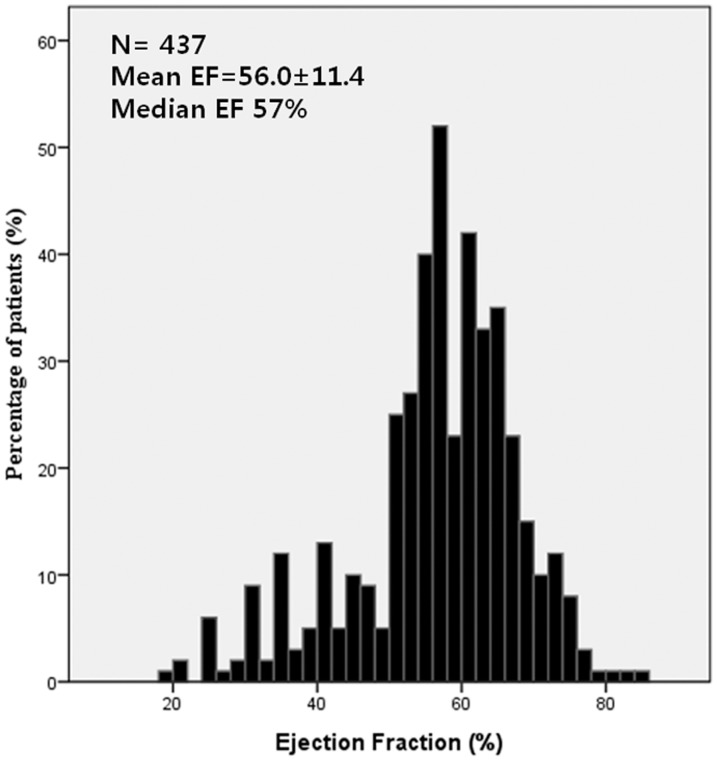
Distribution of EF in included CES patients. Abbreviations: EF:Ejection fraction, CES: Cardioembolic stroke.

Of 2,543 patients registered in SNUHSR between January 2002 and December 2010, 467 patients with CES were initially enrolled. Thirty patients were excluded from this study: 20 with transient ischemic symptom and 10 without documented EF. Finally, a total of 437 patients were included in this study. (Figure 1) The median age of the patients was 69 years (IQR, 61–76) and 57.4% were male. Patients in the subgroup with AF were older, had a higher vascular risk burden and presented with more severe deficits than those without AF. (Table 1) The mean EF was 56.02±11.40% (median EF 57.0%). The distribution of EF is shown in Figure 2. Patients with decreased EF were predominantly male, but prevalence of vascular risk factors and stroke severity were similar between groups. (Table S1 in File S1).

**Table 1:** Basic demographics.

	Total (n = 437)	Subgroup with AF	Subgroup without AF	P value
		(n = 334)	(n = 103)	
Age (years)	67.2±12.9	69.7±10.8	59.2±15.8	<0.001
Male	251 (57.4)	182 (54.5)	69 (67.0)	0.025
Previous stroke	88 (20.1)	72 (21.6)	16 (18.2)	0.183
Hypertension	257 (58.8)	214 (64.1)	43 (41.7)	<0.001
Diabetes	115 (26.3)	92 (27.5)	23 (22.3)	0.293
Hyperlipidemia	79 (18.1)	62 (18.6)	17 (21.5)	0.635
Smoking	100 (22.9)	71 (21.3)	29 (28.2)	0.145
Initial NIHSS				0.006
<7	248 (56.8)	177 (53.0)	71 (68.9)	
7–14	89 (20.4)	78 (23.4)	11 (10.7)	
>14	100 (22.9)	79 (23.7)	21 (20.4)	
IV or IA thrombolysis	81 (18.5)	64 (19.2)	17 (16.5)	0.544
Discharge Warfarin	344 (78.7)	275 (82.3)	69 (67.0)	0.001
Hemorrhagic transformation	92 (21.1)	80 (24.0)	12 (11.7)	0.007

Values are mean±SD or number of patients (percentage).

AF: Atrial fibrillation, NIHSS: National Institutes of Health Stroke Scale, IV: Intravenous, IA: Intraarterial,

In terms of CES etiology, atrial dysrhythmia without structural heart disease was documented in 232 (53.1%) patients, structural heart disease with sinus rhythm in 71 (16.2%) of patients, and combined disease in 104 (23.8%) patients. The remaining 30 (6.8%) patients showed other etiologies: 3 with LA myxoma, 5 with LA thrombus, 5 with LV thrombus and 16 with PFO. The most frequent cardiac source of embolism was AF (76.4%), followed by systolic HF (11.6%). Detailed etiologies of CES are shown in Table S2 in File S1.

### Functional outcome at discharge and short-term mortality

**Figure 3:**
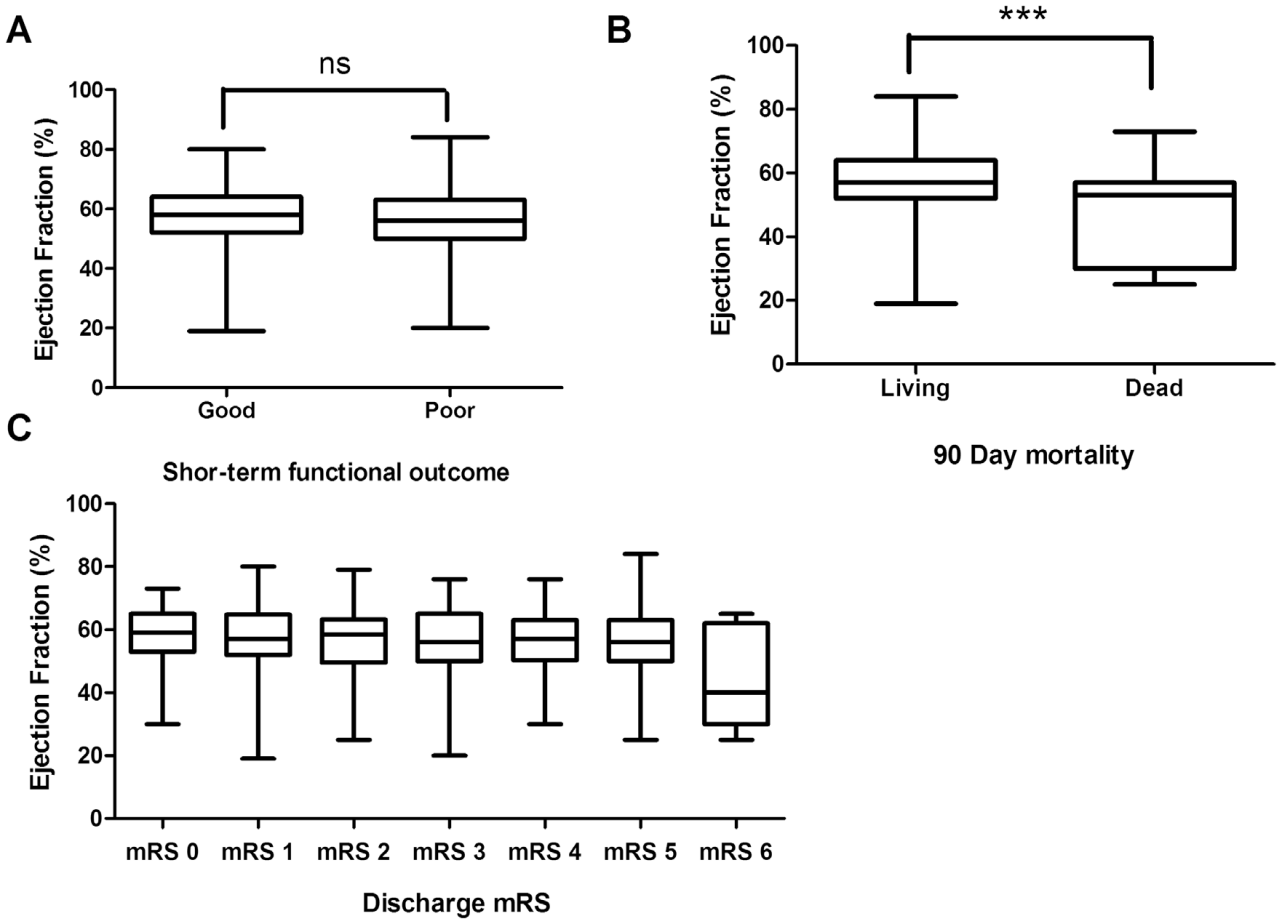
EF according to short-term functional outcome (A), 90 days mortality (B), and mRS in CES patients (C). ns: not significant, ***: p-value <0.01. Abbreviations: ns: not significant, EF, Ejection fraction.mRS: modified Rankin Scale, CES: Cardioembolic stroke.

The median mRS score at discharge was 2 (IQR, 1–4) in CES patients and in the AF subgroup. All patients were followed up in OPD clinic at 3 months and a total 165 patients (37.8%) had poor short-term outcome. While EF was not significantly different according to the discharge mRS (p-value 0.124), EF tended to be lower in patients with poor short-term outcome (56.8±11.0 vs. 54.8±12.0, p-value 0.086). Twenty seven CES patients (6.2%) died within 90 days after admission and EF was significantly higher in the living group. (Figure 3) EF was able to predict 90 day mortality (adjusted OR 0.94) in the multivariate analysis. Older age (adjusted OR = 1.05), initial stroke severity (adjusted OR = 13.18) and warfarin after discharge (OR 0.33) were also associated with 90 day mortality. In the AF subgroup analysis, the 90 day mortality rate was 6.3% and EF showed a tendency to be associated with the 90 day mortality (adjusted OR 0.96), along with the initial stroke severity (adjusted OR 12.63). Age (adjusted OR 1.06) diabetes (adjusted OR 3.42) and warfarin by discharge (adjusted OR 0.35) were independently associated with the 90 day mortality. (Table 2)

**Table 2:** Univariate and Multivariate analysis for 90 days mortality.

	CES patients (27/437)				CES patients with AF (21/334)			
	OR	P	Adjusted OR	P	OR	P	Adjusted OR	P
	(95% CI)		(95% CI)		(95% CI)		(95% CI)	
**EF**	**0.94 (0.91**–**0.97)**	**<0.001**	**0.94 (0.91**–**0.98)**	**0.001**	**0.94 (0.91**–**0.98)**	**0.001**	**0.96 (0.92**–**1.00)**	**0.051**
Age	1.07 (1.03–1.11)	0.001	1.05 (1.01–1.11)	0.028	1.08 (1.03–1.13)	0.003	1.06 (1.00–1.13)	0.042
Sex (Male)	0.92 (0.42–2.02)	0.838			0.91 (0.38–2.21)	0.841		
Previous stroke	0.68 (0.23–2.01)	0.479			0.37 (0.08–1.61)	0.183		
Hypertension	1.20 (0.54–2.70)	0.651			0.91 (0.36–2.25)	0.831		
Diabetes	2.38 (1.08–5.26)	0.031	2.10 (0.78–5.53)	0.144	3.15 (1.29–7.70)	0.012	3.42 (1.14–10.24)	0.028
Hyperlipidemia	0.55 (0.16–1.87)	0.338			0.44 (0.10–1.96)	0.283		
Smoking	0.40 (0.12–1.37)	0.145			0.60 (0.17–2.10)	0.424		
Initial NIHSS		<0.001		0.008		0.002		0.077
<7	1.00 (Reference)		1.00 (Reference)		1.00 (Reference)		1.00 (Reference)	
7–14	10.50 (2.14–51.55)	0.004	6.10 (1.08–34.50)	0.041	17.35 (2.1043.60)	0.008	8.01 (0.88–73.00)	0.065
>14	27.00 (6.13–118.86)	<0.001	13.18 (2.48–70.08)	0.002	34.67(4.45270.24)	0.001	12.63 (1.40–114.34)	0.024
IV or IA thrombolysis	5.51 (2.48–12.26)	<0.001	2.44 (0.78–6.55)	0.077	5.40 (2.18–13.35)	<0.001	2.18 (0.74–6.37)	0.156
Discharge Warfarin	0.13 (0.06–0.30)	<0.001	0.33 (0.12–0.88)	0.027	0.11 (0.04–0.27)	<0.001	0.35 (0.12–1.08)	0.067
Hemorrhagic transformation	3.30 (1.49–7.33)	0.003	0.76 (0.27–2.08)	0.588	2.06 (0.82–5.17)	0.123		

CES: cardioembolic stroke, AF: Atrial fibrillation, OR: Odds Ratio, CI: Confidence Interval, EF: Ejection Fraction,NIHSS: National Institutes of Health Stroke Scale, IV: Intra-venous, IA: Intra-artery.

### Long-term mortality

Overall cumulative death was136 (31.1%) in all CES patients and 106 (31.7%) in the AF subgroup. The relationship between EF and long-term mortality is shown in Table 3. In a multivariable model adjusted for possible covariates, the hazard ratio for mortality significantly decreased by 3% for every 1% increase in ejection fraction in CES patients and 2% for every 1% increase in the AF subgroup. Older patients and those with severe stroke had higher mortality. Patients with hyperlipidemia and statin prescription were protective of long-term cumulative death. (Table 3, Table S3 in File S1).

**Table 3:** Multivariable model hazard ratios for long-term outcomes by EF compared with normal values.

	Adjusted HR (95% CI) of long-term mortality	
	CES patients	CES patients with AF
EF (1% increase in EF)	0.97 (0.96–0.99)	0.98 (0.96–1.00)
EF<40%	2.61 (1.64–4.17)	2.30 (1.29–4.11)
EF 40∼54%	1.65 (0.97–2.81)	1.48 (0.78–2.81)
EF 55∼70%	1.00 (Reference)	1.00 (Reference)

Adjusted for age, sex, history of stroke, hypertension, diabetes, dyslipidemia, smoking,

Admission NIHSS (<7, 7–14, >14), IV or IA thrombolysis,discharge warfarin, hemorrhagic transformation.

EF: Ejection Fraction, CES: cardioembolic stroke, AF: Atrial fibrillation, NIHSS: National Institutes of Health Stroke Scale,

IV: Intra-venous, IA: Intra-arterial.

**Figure 4:**
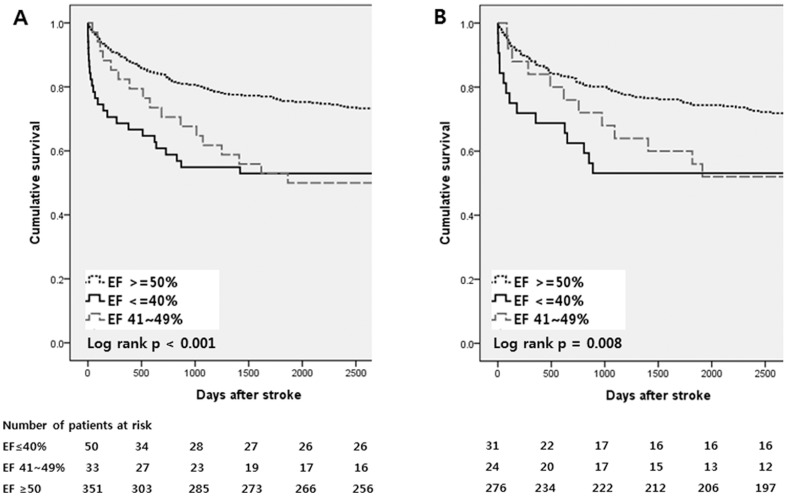
Kaplan-Meier curves of long-term mortality by EF groups in CES patients (A) and AF subgroup (B). Abbreviations: EF, Ejection fraction. CES: Cardioembolic stroke, AF: Atrial fibrillation.

Survival curves are illustrated in Figure 4. Patients with reduced EF (EF≤40%) had a significant higher mortality (HR 2.61) than those with normal EF. Those with borderline EF (41∼49%) had tendency of higher mortality with a lower HR (HR 1.65, p-value 0.067). In the AF subgroup, decreased EF was associated with higher mortality, but with lower HR and less significance. Borderline EF was not associated with mortality. Increasing age and stroke severity were independent predictors of long-term mortality in both CES patient and AF subgroup. Smoking significantly increased mortality in the AF subgroup only. (Table 3, Table S4 in File S1) The mortality data of 34 patients were missing among 136 patients who died. Among others, 38 (37.3%) had vascular death (fatal stroke with ICD code I60–64 or ischemic heart disease caused by myocardiac infarction with ICD code I21–34 as previously defined [19]). Patients with decreased EF had higher HR with vascular death (HR 3.54, p-value 0.003 in CES, HR 2.81, p-value 0.064 in AF subgroup).

## Discussion

Our results show an inverse relationship between EF and long-term cumulative mortality not only in CES patients, but also in the AF subgroup. About 11.7% of our patients had decreased EF (EF≤40%), which is higher than in previous reports (5% to 8%) [4],[5]. Sources of CES were similar compared with previous studies. Decreased EF (EF≤40%) was an important predictor of mortality in patients with CES and in the AF subgroup. Borderline EF of 41∼49% was a poorer predictor of mortality in CES patients and was not predictive of mortality in the AF subgroup. To the best of our knowledge, this is the first study evaluating the relationship of EF as a full spectrum on functional outcomes after CES.

Our data revealed a high association between EF and 90 days mortality which is in line with previous studies. Low EF was identified as an independent predictor of higher short-term mortality in some studies. In a single center study with 503 stroke patients, systolic HF (symptoms and signs of HF and EF<50%) was independently associated with poor outcome (mRS from 3 to 6) in 90 days with an odds ratio of 3.01 [18]. In a study with 130 stroke patients who received thrombolysis, decreased EF (EF<40%) was independently associated with a higher 90-day mortality rate (OR 9.88) along with the clinical diagnosis of HF (OR 4.17) [5]. However, there are conflicting results with regard to the relationship between reduced EF and long-term mortality. A recent publication, evaluating stroke patients with EF≤35%revealed low EF is an independent predictor of 1 year mortality along with increasing age, stroke severity and AF [6]. In contrast, in a study with 283 stroke patients with HF, cumulative mortality up to 10 years was not associated with low EF (EF<40%), but with the severity of heart failure and stroke [4]. Although these studies evaluated a direct association between low EF and long-term mortality after stroke, they are limited by several factors. First, they included stroke patients with heterogenous subtypes. Those with lower EF may have a higher prevalence of CES, which can distort the true relationship between EF and poor outcome. CES is known to cause highest mortality and worst functional outcome [9], [10]. Our study is unique evaluating patients with single stroke subtype. We found a significant relationship between EF and long-term cumulative mortality in patients with CES and notably in the subgroup with AF. Second, previous studies have arbitrary dichotomized the EF spectrum. There is a lack of consensus with regard to the cut-off value for defining decreased EF and various indicators (EF<40% [5], EF<50% [18], and EF<55% [16]) have been used as a criteria for reduced EF. Dichotomization of EF prevents us from evaluating EF as a whole, especially those with neither preserved nor decreased LVEF (Borderline EF) and can result in a biased outcome. We applied EF as both continuous and categorical variable to evaluate a true association between EF and outcome after CES and found a significant association even when possible covariates were adjusted. We also evaluated EF as a categorical variable to further evaluate the true relation between EF and stroke outcome. As expected, the association between reduced EF≤40% and long-term mortality was high. Patients with borderline EF (41∼49%) showed a tendency of higher mortality than patients in the normal EF group, which was neglected in the previous studies. This reinforces the concept that lower EF is related with a higher mortality in CES. In the AF subgroup, decreased EF had a weaker association with long-term mortality compared with total CES patients. AF itself is known to cause greater disability and mortality in stroke patients [20], [21]. In our study population, patients with AF had higher vascular risk burden and worse deficits than non-AF patients. Influence of AF may have masked the effect of lower EF on mortality. Nonetheless, it is important to note that low EF is an independent predictor of long-term mortality even in the AF subgroup.

There are several explanations for a poor outcome after stroke in patients with decreased cardiac function. Patients with decreased EF have elevated LV filling pressure, causing decreased stroke volume. Moreover, decreased EF impairs cerebrovascular reactivity, compromising cerebral autoregulation, eventually causing chronic cerebral hypoperfusion [22], [23]. Some investigators explain that patients with low EF are complicated with other co-morbidities that increase atherosclerotic burden, though this was not significant in our study.

There are some limitations in our study. First, this study is a single-center study and our result has to be confirmed in other populations. Also Stroke recurrence was not evaluated in this study as a common complication among CES patients causing high mortality [2]. Although we included previous stroke as a covariate in the analysis, to document stroke recurrence after enrollment was impossible through registry data. Cause of death was not properly evaluated. Even though mortality data are partial, a higher HR of decreased EF on vascular mortality suggests more CES patients with decreased EF died due to vascular causes. In addition, baseline functional status was not considered in our study and the discharge mRS data were used as short-term outcome instead of the 3 months mRS. Other inevitable limitations are possible selection bias of a single center study and the possibility of missing confounding factors while applying multivariate logistic analysis.

## Conclusions

A strong association was found between lower EF and outcome after CES. As expected, decreased EF (EF≤40%) had a strong relationship with higher mortality. Patients with borderline EF (EF 41∼49%) showed a tendency of higher mortality which has not been evaluated in previous studies. This result was independent of patient age, sex, vascular risk factors, stroke severity, in-hospital management and hemorrhagic transformation. Moreover, we found associations in the subgroup of patients with AF, which are known to have higher mortality. No significant association was found between borderline EF and long-term mortality in the AF subgroup. Taken together, our study suggests the importance of echocardiographic evaluation in CES patients, even in patients with AF.

## Supporting Information

File S1 Contains Table S1, baseline characteristics by EF groups in CES. Table S2, etiology of CES. Table S3, adjusted hazard ratio for long-term mortality. Table S4, multivariable model hazard ratios for long-term outcomes.
(DOC)
