# Supplementary material for: Cardiac Function and Outcome in Patients with Cardio-Embolic Stroke
Source: PLoS One. 2014 Apr 23;9(4):e95277. doi: 10.1371/journal.pone.0095277 (PMC3997393; doi:10.1371/journal.pone.0095277)
Supplement: File S1 — Contains Table S1, baseline characteristics by EF groups in CES. Table S2, etiology of CES. Table S3, adjusted hazard ratio for long-term mortality. Table S4, multivariable model hazard ratios for long-term outcomes. (DOC) [file pone.0095277.s001.doc]

**Table S1. Baseline characteristics by EF groups in CES**

|  | **EF≤40%**  **(n=51)** | **EF 41~49%**  **(n=34)** | **EF ≥50%**  **(n=352)** | **P-value** |
| --- | --- | --- | --- | --- |
| Age | 66.7±13.7 | 67.4±15.1 | 67.3±12.6 | 0.952 |
| Sex (Male) | 37 (72.5) | 21 (61.8) | 193 (54.8) | 0.050 |
| Previous stroke | 13 (25.5) | 3 (8.8) | 72 (20.5) | 0.162 |
| Hypertension | 26 (51.0) | 20 (58.8) | 211 (59.9) | 0.478 |
| Diabetes | 18 (35.3) | 9 (26.5) | 88 (25.0) | 0.296 |
| Hyperlipidemia | 9 (17.6) | 6 (17.6) | 64 (18.2) | 0.993 |
| Smoking | 12 (23.5) | 7 (20.6) | 81 (23.0) | 0.943 |
| Initial NIHSS |  |  |  | 0.222 |
| <7 | 22 (51.2) | 55 (50.9) | 153 (60.0) |  |
| 7-14 | 8 (18.6) | 24 (22.2) | 54 (21.2) |  |
| >14 | 13 (30.2) | 29 (26.9) | 48 (18.8) |  |
| IV or IA thrombolysis | 9 (11.1) | 10 (29.4) | 62 (17.6) | 0.236 |
| Discharge Warfarin | 36 (70.6) | 27 (79.4) | 281 (79.8) | 0.320 |
| Hemorrhagic transformation | 14 (27.5) | 9 (26.5) | 69 (19.6) | 0.316 |

Values are mean±SD or number of patients (percentage).

EF: Ejection Fraction, NIHSS: National Institutes of Health Stroke Scale, IV: Intravenous, IA: Intraarterial

**Table S2**. Etiology of CES

| **Total** | **n = 437** |
| --- | --- |
| **Arrythmia without structural heart disease** | **232 (53.1%)** |
| Atrial fibrillation | 189 |
| Paroxysmal Atrial fibrillation | 38 |
| Atrial flutter | 1 |
| Sick sinus syndrome | 4 |
|  |  |
| **Isolated structural heart disease** | **71 (16.2%)** |
| Ischemic heart disease | 37 |
| -Congestive Heart Failure | 16 |
| -LV akinesia more than two segments | 16 |
| -LV Aneurysm | 1 |
| -MI (Acute MI or Old MI) | 4 |
| Dilated Cardiomyopathy | 7 |
| Infective endocarditis | 5 |
| Prosthetic valve | 20 |
| Atrial septal Aneurysm | 1 |
| Valvular disease | 1 |
|  |  |
| **Arrythmia with structural heart disease** | **104 (23.8%)** |
| Atrial fibrillation | 86 |
| Paroxysmal Atrial fibrillation | 17 |
| Atrial flutter | 1 |
|  |  |
| Ischemic heart disease | 61 |
| -Congestive Heart Failure | 35 |
| -LV akinesia more than two segments | 14 |
| -LV Aneurysm | 1 |
| -MI (Acute MI or Old MI) | 11 |
| Dilated Cardiomyopathy | 6 |
| Prosthetic valve | 16 |
| Valvular disease | 21 |
|  |  |
| **Others** | **30 (6.8%)** |
| LA myxoma | 3 |
| LA thrombus | 5 |
| LV thrombus | 5 |
| PFO | 16 |
| Pulmonary AVM | 1 |

CES: Cardioembolic stroke, LV: Left Ventricle, MI: Myocardiac infarction, PFO: Patent foramen ovale, AVM: Arteriovenous malformation

| **Table S3. Adjusted hazard ratio for long-term mortality** | | | | | |
| --- | --- | --- | --- | --- | --- |
|  | **CES patients (136/437)** | |  | **CES patients with AF** | |
|  | **Adjusted HR (95% CI)** | **P value** |  | **Adjusted HR (95% CI)** | **P value** |
| **EF** | **0.97 (0.96-0.99)** | **0.001** |  | **0.98 (0.96-1.00)** | **0.031** |
| Age | 1.04 (1.03-1.06) | <0.001 |  | 1.04 (1.02-1.06) | <0.001 |
| Sex (Female) | 1.07 (0.72-1.58) | 0.737 |  | 0.89 (0.57-1.38) | 0.597 |
| Previous stroke | 1.08 (0.71-1.65) | 0.725 |  | 1.21 (0.76-1.93) | 0.414 |
| Hypertension | 1.12 (0.77-1.62 | 0.562 |  | 1.06 (0.68-1.63) | 0.810 |
| Diabetes | 0.76 (0.50-1.15) | 0.194 |  | 0.99 (0.63-1.56) | 0.956 |
| Hyperlipidemia | 0.56 (0.33-0.97) | 0.038 |  | 0.48 (0.26-0.91) | 0.024 |
| Smoking | 1.38 (0.88-2.15) | 0.158 |  | 1.61 (0.96-2.70) | 0.069 |
| Initial NIHSS |  | <0.001 |  |  | <0.001 |
| <7 | 1.00 (Reference) |  |  | 1.00 (Reference) |  |
| 7-14 | 2.02 (1.26-3.21 | 0.003 |  | 2.15 (1.25-3.69) | 0.005 |
| >14 | 3.33 (2.03.01) | <0.001 |  | 3.47 (2.02-5.95) | <0.001 |
| IV or IA thrombolysis | 0.74 (0.47-1.16) | 0.186 |  | 0.65 (0.39-1.10) | 0.106 |
| Discharge Warfarin | 0.75 (0.50-1.11) | 0.149 |  | 0.73 (0.45-1.17) | 0.190 |
| Hemorrhagic transformation | 1.17 (0.79-1.75) | 0.437 |  | 1.23 (0.79-1.93) | 0.365 |

CES: cardioembolic stroke, AF: Atrial fibrillation, HR: Hazzard Ratio, CI: Confidence Interval, EF: Ejection Fraction, NIHSS: National Institutes of Health Stroke Scale, IV: Intra-venous, IA: Intra-arterial

| **Table S4. Multivariable model hazard ratios for long-term outcomes** | | | | | |
| --- | --- | --- | --- | --- | --- |
|  | **CES patients (136/437)** | |  | **CES patients with AF** | |
|  | **Adjusted HR (95% CI)** | **P value** |  | **Adjusted HR (95% CI)** | **P value** |
| **EF** |  | **<0.001** |  |  | **0.014** |
| **≤40%** | **2.61 (1.64-4.17)** | **<0.001** |  | **2.30 (1.29-4.11)** | **0.005** |
| **41~49%** | **1.65 (0.97-2.81)** | **0.067** |  | **1.48 (0.78-2.81)** | **0.229** |
| **≥50%** | **1.00 (Reference)** |  |  | **1.00 (Reference)** |  |
| Age | 1.04 (1.03-1.06) | <0.001 |  | 1.04 (1.02-1.07) | <0.001 |
| Sex (Male) | 1.03 (0.70-1.53) | 0.871 |  | 0.85 (0.54-1.33) | 0.485 |
| Previous stroke | 1.06 (0.69-1.61) | 0.805 |  | 1.19 (0.74-1.90) | 0.469 |
| Hypertension | 1.09 (0.75-1.59) | 0.644 |  | 1.05 (0.68-1.61) | 0.840 |
| Diabetes | 0.77 (0.51-1.60) | 0.208 |  | 0.98 (0.62-1.55) | 0.932 |
| Hyperlipidemia | 0.56 (0.33-0.96) | 0.036 |  | 0.47 (0.25-0.89) | 0.020 |
| Smoking | 1.44 (0.93-2.25) | 0.106 |  | 1.71 (1.02-2.87) | 0.042 |
| Initial NIHSS |  | <0.001 |  |  | <0.001 |
| <7 | 1.00 (Reference) |  |  | 1.00 (Reference) |  |
| 7-14 | 2.14 (1.34-3.41) | 0.001 |  | 2.16 (1.36-3.69) | 0.005 |
| >14 | 3.33 (2.12-5.24) | <0.001 |  | 3.45 (2.02-5.92) | <0.001 |
| IV or IA thrombolysis | 0.74 (0.47-1.17) | 0.204 |  | 0.67 (0.40-1.31) | 0.135 |
| Discharge Warfarin | 0.78 (0.53-1.17) | 0.231 |  | 0.75 (0.46-1.21) | 0.241 |
| Hemorrhagic transformation | 1.16 (0.78-1.72) | 0.480 |  | 1.25 (0.79-1.95) | 0.340 |

CES: cardioembolic stroke, AF: Atrial fibrillation, HR: Hazzard Ratio, CI: Confidence Interval, EF: Ejection Fraction, NIHSS: National Institutes of Health Stroke Scale, IV: Intra-venous, IA: Intra-arterial
